# Clinical Decision Support Requirements for Ventricular Tachycardia Diagnosis Within the Frameworks of Knowledge and Practice: Survey Study

**DOI:** 10.2196/55802

**Published:** 2024-03-26

**Authors:** Zhao Hu, Min Wang, Si Zheng, Xiaowei Xu, Zhuxin Zhang, Qiaoyue Ge, Jiao Li, Yan Yao

**Affiliations:** 1 Arrhythmia Center Fuwai Hospital Chinese Academy of Medical Sciences & Peking Union Medical College/National Center for Cardiovascular Diseases Beijing China; 2 Institute of Medical Information Chinese Academy of Medical Sciences & Peking Union Medical College Beijing China; 3 West China School of Public Health West China Fourth Hospital Sichuan University Chengdu China

**Keywords:** clinical decision support system, requirements analysis, ventricular tachycardia, knowledge, clinical practice, questionnaires

## Abstract

**Background:**

Ventricular tachycardia (VT) diagnosis is challenging due to the similarity between VT and some forms of supraventricular tachycardia, complexity of clinical manifestations, heterogeneity of underlying diseases, and potential for life-threatening hemodynamic instability. Clinical decision support systems (CDSSs) have emerged as promising tools to augment the diagnostic capabilities of cardiologists. However, a requirements analysis is acknowledged to be vital for the success of a CDSS, especially for complex clinical tasks such as VT diagnosis.

**Objective:**

The aims of this study were to analyze the requirements for a VT diagnosis CDSS within the frameworks of knowledge and practice and to determine the clinical decision support (CDS) needs.

**Methods:**

Our multidisciplinary team first conducted semistructured interviews with seven cardiologists related to the clinical challenges of VT and expected decision support. A questionnaire was designed by the multidisciplinary team based on the results of interviews. The questionnaire was divided into four sections: demographic information, knowledge assessment, practice assessment, and CDS needs. The practice section consisted of two simulated cases for a total score of 10 marks. Online questionnaires were disseminated to registered cardiologists across China from December 2022 to February 2023. The scores for the practice section were summarized as continuous variables, using the mean, median, and range. The knowledge and CDS needs sections were assessed using a 4-point Likert scale without a neutral option. Kruskal-Wallis tests were performed to investigate the relationship between scores and practice years or specialty.

**Results:**

Of the 687 cardiologists who completed the questionnaire, 567 responses were eligible for further analysis. The results of the knowledge assessment showed that 383 cardiologists (68%) lacked knowledge in diagnostic evaluation. The overall average score of the practice assessment was 6.11 (SD 0.55); the etiological diagnosis section had the highest overall scores (mean 6.74, SD 1.75), whereas the diagnostic evaluation section had the lowest scores (mean 5.78, SD 1.19). A majority of cardiologists (344/567, 60.7%) reported the need for a CDSS. There was a significant difference in practice competency scores between general cardiologists and arrhythmia specialists (*P*=.02).

**Conclusions:**

There was a notable deficiency in the knowledge and practice of VT among Chinese cardiologists. Specific knowledge and practice support requirements were identified, which provide a foundation for further development and optimization of a CDSS. Moreover, it is important to consider clinicians’ specialization levels and years of practice for effective and personalized support.

## Introduction

Sudden cardiac death (SCD) remains a significant public health issue, accounting for 50% of all cardiovascular deaths. The estimated annual incidences of SCD are 60 [1], 40.7 [2,3], and 36.8 [4] per 100,000 people in the United States, China, and Europe, respectively. Ventricular tachycardia (VT) is a major cause or precursor of SCD [5], which can be the initial or sole manifestation of diverse heart diseases [6,7]. VT diagnosis is challenging due to its similarity with some forms of supraventricular tachycardia, the complexity of clinical manifestations, heterogeneity of underlying diseases, and potential for life-threatening hemodynamic instability [6,8]. Diagnostic accuracy and timing are critical for patients with VT, as the stage of diagnosis determines the selection of treatment [9]. However, studies have revealed a substantial prevalence of misdiagnoses of VT [10-13], focusing on differential diagnosis between VT and supraventricular tachycardia. Although diagnostic error has been a challenge along the development of medicine, measuring diagnostic error can be difficult due to detection and reporting biases, with scarce reports indicating error rates of approximately 10%-15% [14]. We could not find additional estimates for the actual diagnostic error of VT; however, it is commonly acknowledged to represent a substantial challenge considering the complexity of the condition [9,15].

Diagnosis represents a complex cognitive process comprising a variety of different problem-solving tasks that are related to the clinical reasoning process, such as taking a medical history, forming a differential diagnosis, ordering examinations, and interpreting clinical findings [16]. The diagnostic process requires not only the retention of knowledge but also the judicious application of that knowledge at opportune moments, namely in clinical practice. A proper diagnosis of VT demands a great volume of knowledge. First, the clinician must be able to identify VT among the spectrum of wide QRS tachycardias by inspecting a list of electrocardiogram (ECG) features and comparing the findings to various diagnostic criteria or algorithms [17,18]. Once VT is identified by ECG interpretation, the next step is to diagnose the underlying diseases from a vast disease spectrum. This is a particularly challenging task, as any disease involving the myocardium can cause VT, such as coronary artery disease (CAD), all types of cardiomyopathies, myocarditis, inherited arrhythmia syndromes, autoimmune or inflammatory diseases, and others [7,9]. Moreover, translating the enormous body of knowledge into proper practice can be difficult [19], which is exacerbated by the fact that VT can cause stress to clinicians due to the probability of hemodynamic instability.

In response to this challenge, the clinical decision support system (CDSS) has emerged as a promising tool to augment the diagnostic capabilities of clinicians. Clinical decision support (CDS) is a process for enhancing health-related decisions with pertinent, organized clinical knowledge and patient information, thus advancing health care delivery [20]. Use of a CDSS can provide clinicians with situation-specific knowledge that aids in making critical clinical decisions such as risk assessment, diagnosis, prognosis, and selection of therapy [21]. A clinical diagnostic decision support system (DDSS) is a computer-based algorithm that assists a clinician with one or more component steps of the diagnostic process [22]. A DDSS is expected to receive relevant patient information and return outputs to assist with the problems the clinician has encountered in the diagnostic process, such as suggesting a likely diagnosis. Some well-known DDSSs such as ISABEL [23] and Dxplain [24] provide a diagnosis list, which can offer a solution to the challenges associated with VT diagnosis. Most CDSSs exhibit efficacy in a laboratory or experimental environment; however, relatively few such systems are being used at present and the rate of use in routine clinical practice is low [20,25-27]. Studies have identified the main barriers to the widespread adoption of CDSSs, including vague requirements, poor integration with the clinical workflow, low user acceptance or trust, and lack of transparency. Among these barriers, comprehensive user requirements engineering should be performed at the very beginning of development, which should be continued iteratively throughout the CDSS design-development-implementation life cycle [25,26,28,29]. To address this gap, several recent studies have aimed at elucidating the clinical requirements for an effective and usable CDSS in the context of specific fields or scenarios [30-34] with a variety of methods, including focus groups [30,35], a workshop [34], expert discussion with a literature review [36,37], semistructured interviews [31,34,35,38], writing user stories [39], and system evaluation [40]. Overall, most studies have adopted a user-centered approach with qualitative analysis.

To our best knowledge, although an artificial intelligence model was reported for predicting the in-hospital mortality of VT [8], no CDSS has been developed for VT diagnosis. A recent systemic review of cardiovascular CDSSs found that the complexity of the clinical management of cardiovascular disease itself was a barrier during implementation [27], which emphasizes the need for an authentic clinical requirements analysis. Accordingly, the objective of this study was to analyze the requirements for a VT diagnosis CDSS within the frameworks of knowledge, practice, and CDS needs.

## Methods

### Study Design and Recruitment Process

**Figure 1:**
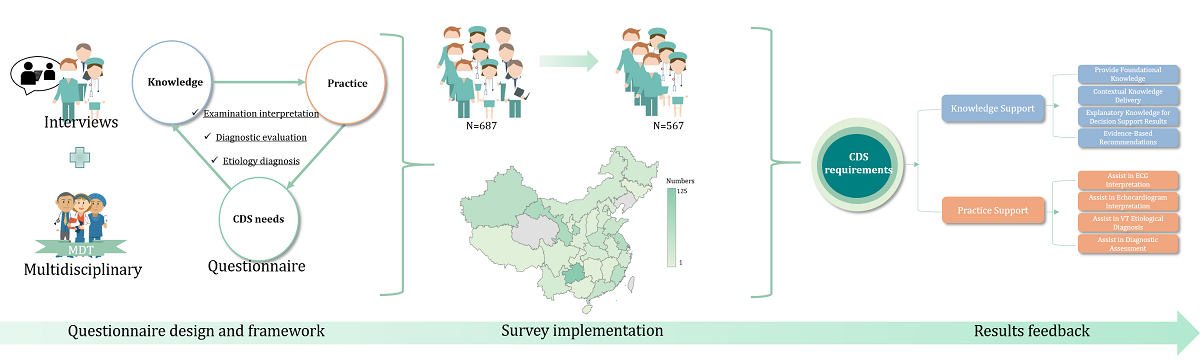
Schematic of the overall study workflow and assessment approach. CDS: clinical decision support; ECG: electrocardiogram; MDT: multidisciplinary team; VT: ventricular tachycardia.

Figure 1 shows the overall flow of our study, which consisted of semistructured interviews in the early stages and questionnaires in the later stages. To effectively implement and conduct the questionnaire assessment, we conducted open and explorative interviews about the challenges associated with the management of VT and the expected functions of a CDSS for VT. The interviews were conducted at Fuwai Hospital, the national cardiovascular disease center of China. This hospital actively recruits cardiologists for their fellowships from all regions of China, resulting in a representative sample of interviewees. We sent interview invitations to all 56 cardiologists in the arrythmia center, including cardiologists from the fellowship program or established staff of Fuwai Hospital. Seven cardiologists responded and completed the interview, followed by a brief questionnaire to provide information on demographics and clinical experience (see Multimedia Appendix 1).

A multidisciplinary team was formed to define the purpose of our study and the design of the questionnaire based on the interview results. The multidisciplinary team comprised three arrhythmia specialists, three experts in medical informatics and CDS, and one clinical statistician. The questionnaire was examined by an additional 20 arrhythmia specialists to ensure its clarity and feasibility. We conducted a nationwide cross-sectional survey with an online questionnaire in mainland China from December 31, 2022, to February 15, 2023. We recruited registered cardiologists using a convenience sampling approach from network groups associated with the Asian Heart Rhythm Association (AHRA) on WeChat, the dominant social media app in China. The AHRA is an academic organization focusing on arrhythmias, whose members are all registered cardiologists. Duplicate submissions were prevented through IP address constraints, and only completed responses were included for analysis.

### Ethical Considerations

Participants provided online informed consent, which detailed the survey’s background, aim, methods, and confidentiality measures. To protect participants’ privacy, a signature was not required. Instead, participants clicked the “go on” button at the bottom of the informed consent page if they agreed to participate. According to data privacy protocols, no personal information, including the participants’ names or affiliations, was collected. Since patients were not the subject of this study, ethical approval was exempted by the ethics committee of the Institute of Medical Information, Chinese Academy of Medical Sciences/Peking Union Medical College [41]. Each participant received ~US $3 as compensation.

### Questionnaire Design

#### Overview

The questionnaire was divided into four sections (Table 1): demographic information (questions 1-6), knowledge assessment (question 14), practice assessment (questions 7-13), and CDS needs (questions 15-18). A comprehensive version of the questionnaire is provided in Multimedia Appendix 2.

**Table 1:** Design of the questionnaire.

Section	Content	Related questions
Knowledge	Examination interpretation, etiological diagnosis, diagnostic evaluation, conceptual knowledge	14
Practice	Examination interpretation, etiological diagnosis, diagnostic evaluation	7-13
Clinical decision support needs	Interpretable diagnosis, executable processes, knowledge support	15-18

#### Knowledge Assessment

Knowledge serves as the theoretical foundation for clinicians to make clinical diagnoses and is thus an essential competency for clinicians. The diagnosis of VT is difficult as it will largely depend on the clinician’s familiarity with the vast knowledge of the field. The European Society of Cardiology (ESC) guideline suggests a protocol for VT diagnosis [15]. The multidisciplinary team abstracted the knowledge points from the ESC guideline for collecting information on the participants’ self-reported knowledge shortcomings.

#### Practice Assessment

##### Areas of Focus

To attain a more accurate gauge of the clinical practice competency, we used simulated cases rather than straightforward questions [42], which can help differentiate practice competency from knowledge. To mitigate the risk of low response rates and careless submissions associated with lengthy surveys [43], we designed two stepwise cases containing seven questions. According to the intention, the questions about clinical practice were divided into three parts: examination interpretation, etiological diagnosis, and diagnostic evaluation. Multiple-choice options were available for all the questions. We standardized the total score for each section to 10 points according to the weighting.

##### Examination Interpretation

Accurate interpretation of an examination is the basis for a correct etiological diagnosis. ECG is the first-line examination modality for arrhythmias, as nearly all arrhythmia episodes are detected by ECG. Therefore, for this section, we focused on the identification of VT and sites of origin of VT on ECG [15].

##### Etiological Diagnosis

A correct etiological diagnosis of VT is necessary for appropriate treatment. The main strategy is to identify or exclude structural heart diseases, including CAD, myocarditis, and cardiomyopathies [44]. In this section, we assessed the correctness of a diagnosis of arrhythmogenic right ventricular cardiomyopathy (ARVC) and acute myocarditis as the two cases.

##### Diagnostic Evaluation

Diagnostic evaluation is a process of collecting clinical information to confirm or exclude a suspected diagnosis. A diagnostic evaluation protocol for VT is recommended in the ESC guideline [15] with the goal of reducing the rate of diagnostic errors. Based on the cases with an etiological diagnosis, we assessed the competency of the participants to arrange further diagnostic evaluations.

#### CDS Needs

According to the ESC guideline [15] and universal CDSS functionality [25], the multidisciplinary team summarized the results of the interviews to produce a list of functions required for CDS, which could be divided into executable processes, interpretable diagnosis, and knowledge support. We employed this list to poll the functionalities required by the cardiologists for a VT CDSS.

### Quality Control of Responses

To ensure the validity and reliability of our survey responses, we used two strategies to filter out potentially low-quality submissions. First, participants who completed the questionnaire in under 2 minutes were excluded. This threshold was determined through a pretest evaluation coupled with multidisciplinary team discussions. Second, responses were considered to be invalid if participants selected all the available options for questions 7, 8, 9, 11, 12, or 13. This exclusion criterion was established based on the consensus opinion of the multidisciplinary team, who deemed such selections to be unreasonable.

### Statistical Analysis

We only included valid questionnaire responses in the statistical analysis. All data in the demographic section were categorical. Comparisons were performed using mean, median, range, and percentage. The scores in the practice section are expressed as continuous variables, using the mean, median, and range. The knowledge and CDS sections were phrased as single-choice questions asking clinicians about their subjective views on given statements using a 4-point Likert scale without a neutral option. The internal consistency of the questionnaire was assessed using the Cronbach α value.

In addition, we grouped participants separately by practice years and specialty for further subgroup analyses. The Kruskal-Wallis test was performed to investigate the relationship between practice scores and practice years or specialty. All analyses were conducted in R version 4.0.3 [45]. We analyzed most of the data descriptively using graphics produced by the R package ggplot2.

## Results

### Sociodemographic Characteristics of Participants

A total of 687 questionnaires were completed. After applying our quality control measures, 567 responses were considered valid, yielding a validity rate of 82.53%. Among the invalid questionnaires, 104 responses were excluded due to a completion time of less than 2 minutes and 16 were excluded for selecting all options in questions 7, 8, 9, 11, 12, or 13. Descriptive statistics regarding the sociodemographic characteristics of participants are presented in Table 2. Of the enrolled participants, 54.50% were men; 93.47% were general cardiologists and the others were cardiac arrhythmia specialists. More than half of the participants were from tertiary A hospitals. Only a small percentage of cardiologists had ever used a CDSS, and the majority reported needing a CDSS to assist them in the management of VT (Table 2).

**Table 2:** Demographic characteristics of the survey participants (N=567).

Characteristics		Participants, n (%)
**Gender**		
	Woman	258 (45.5)
	Man	309 (54.50)
**Age (years)**		
	≤30	89 (15.7)
	31-35	152 (26.81)
	36-40	129 (22.75)
	41-45	92 (16.23)
	46-50	60 (10.58)
	≥51	45 (7.94)
**Department**		
	Cardiology	530 (93.47)
	Cardiac arrhythmia specialty	39 (6.88)
**Professional title**		
	Resident physician	120 (21.16)
	Attending	237 (41.8)
	Associate chief	145 (25.57)
	Chief	65 (11.46)
**Years of practice**		
	<10	247 (43.54)
	10-20	213 (37.57)
	>20	107 (18.87)
**Hospital tier**		
	Tertiary A	414 (73.02)
	Not tertiary A	153 (26.98)
**Ever used a CDSS^a^?**		
	Yes	72 (12.70)
	No	495 (87.30)
**Is there a need for a CDSS?**		
	Yes	523 (92.24)
	No	44 (7.76)

^a^CDSS: clinical decision support system.

### Semistructured Interviews

Textbox 1 summarizes the results of the semistructured interviews, in which we focused on the challenges of VT management and CDSS needs. The responses of the seven cardiologists were focused, with each noting that etiological diagnosis and interpretation of ECG results were their main challenges. The most important demand was the provision of quick and concise recommendations on diagnosis and treatment. The interviewees also expected the CDSS to provide clinical pathways.

**Results of the interviews.**

- Challenges in the management of ventricular tachycardia (VT)

1. Etiological diagnosis
2. Wide QRS tachycardia diagnosis on electrocardiogram (ECG)
3. Determination of the location of VT origin on ECG
4. Mechanisms of VT
5. Drug treatment options
6. Options for the treatment of polymorphic VT

- Clinical decision support system needs

1. Rapid and concise recommendations for diagnosis and treatment
2. Diagnostic and therapeutic pathways for different etiologies
3. Aids in the identification of wide QRS
4. Adjunctive etiological diagnosis
5. Diagnostic supplements for related diseases

### Knowledge

**Figure 2:**
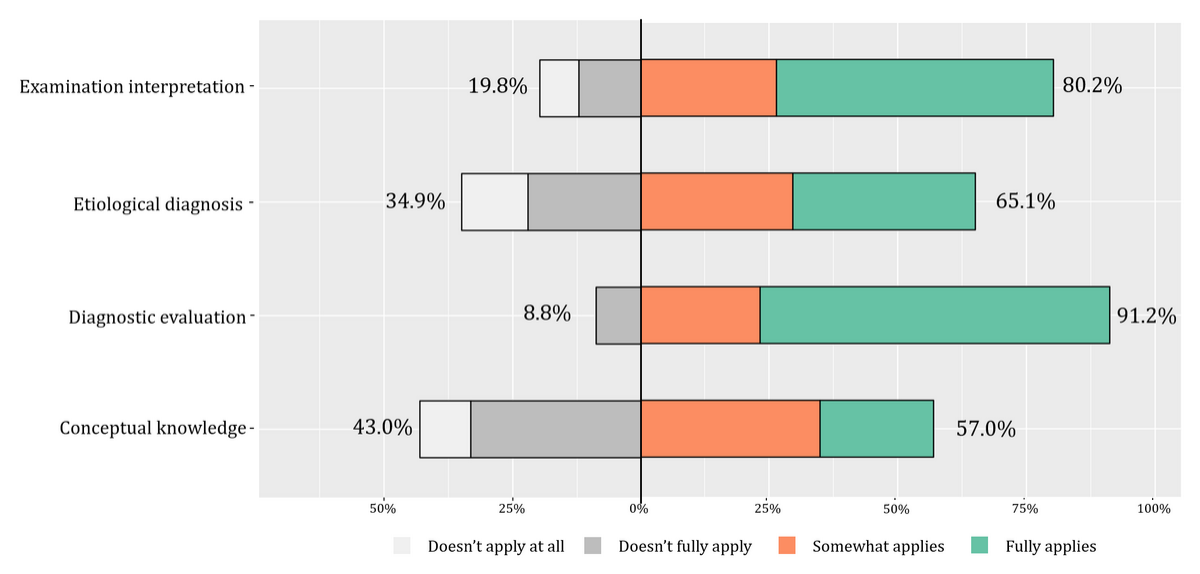
Knowledge assessment.

Figure 2 shows that there was an overall lack of knowledge with respect to diagnostic evaluation, with 383 of the 567 (68.0%) cardiologists indicating full need of assistant knowledge in diagnostic evaluation. This was followed by examination interpretation, where 305 of the 567 (53.8%) cardiologists were in full need of knowledge regarding the interpretation of ECG, cardiac ultrasound, and other cardiac examinations. The need for conceptual knowledge was relatively lower, even though it still reached nearly 60%.

### Practice

**Figure 3:**
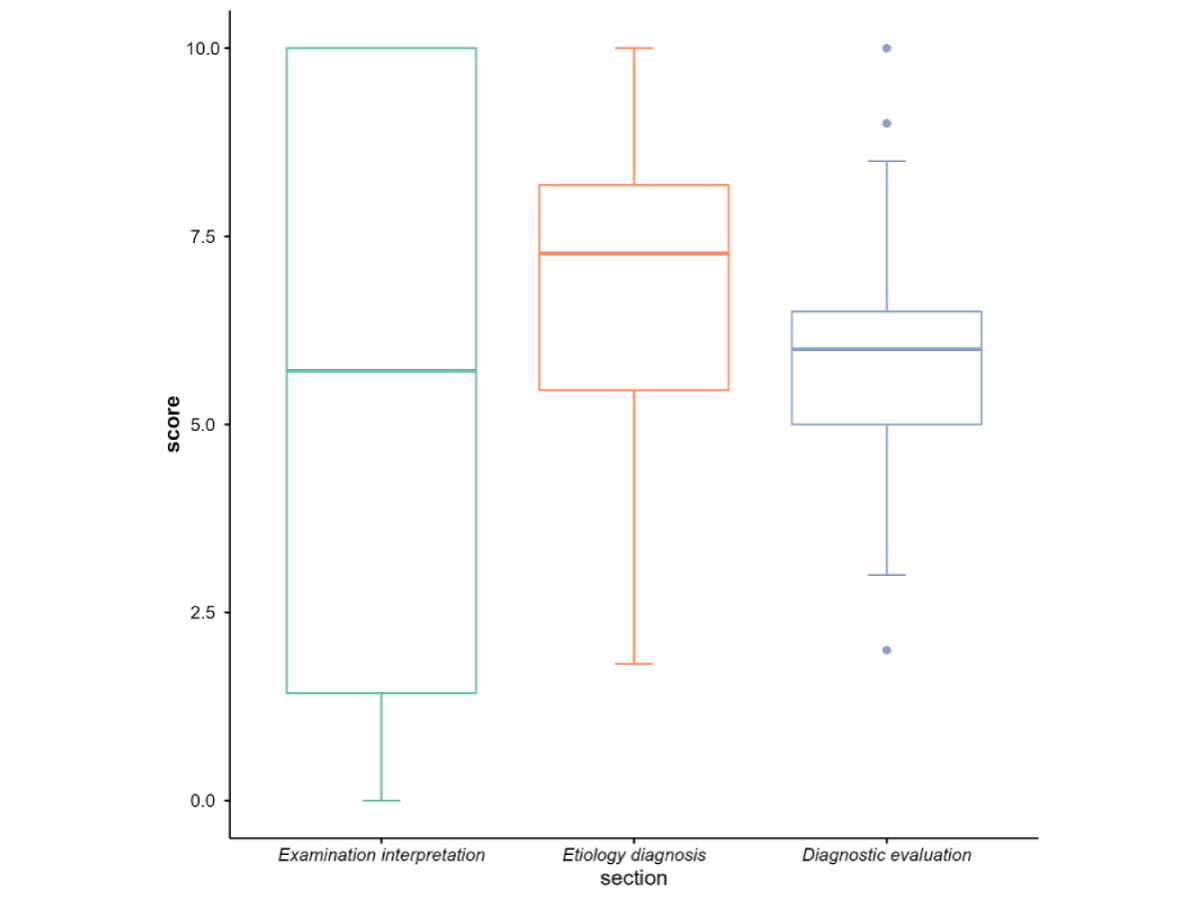
Practice assessment.

The overall average score of the practice questions was 6.11 (SD 0.55), the internal consistency of which was confirmed by a Cronbach α of 0.913. The mean scores of the examination interpretation, etiological diagnosis, and diagnostic evaluation were 6.22 (SD 3.94), 6.74 (SD 1.75), and 5.78 (SD 1.19), respectively. As shown in Figure 3, the etiological diagnosis section was associated with the highest overall score and the distribution of scores was also more concentrated than for the other sections, especially when compared with the distribution of the examination interpretation scores that were more dispersed and polarized.

### CDS Needs

**Figure 4:**
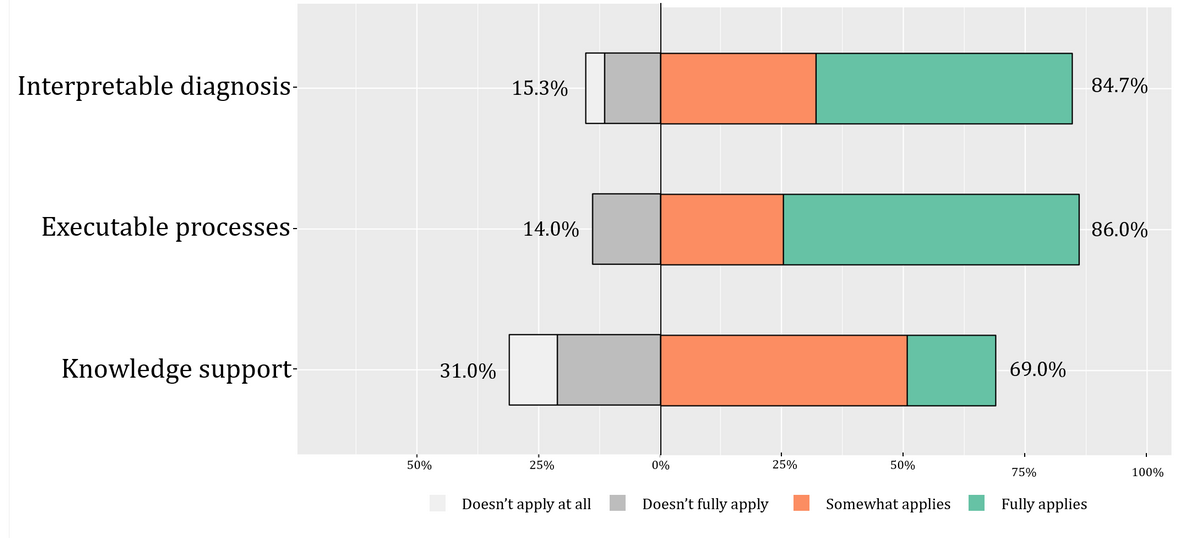
Clinical decision support needs assessment.

The majority of the surveyed cardiologists reported a positive attitude toward CDS needs (Figure 4). There was relatively higher demand expressed for functions related to executable processes and interpretable diagnosis. In particular, the executable processes function was considered to be an essential requirement of a CDSS by 344 of the 567 cardiologists (60.7%). Knowledge support function received the least support but was still close to 70%.

### Subgroup Analysis

**Figure 5:**
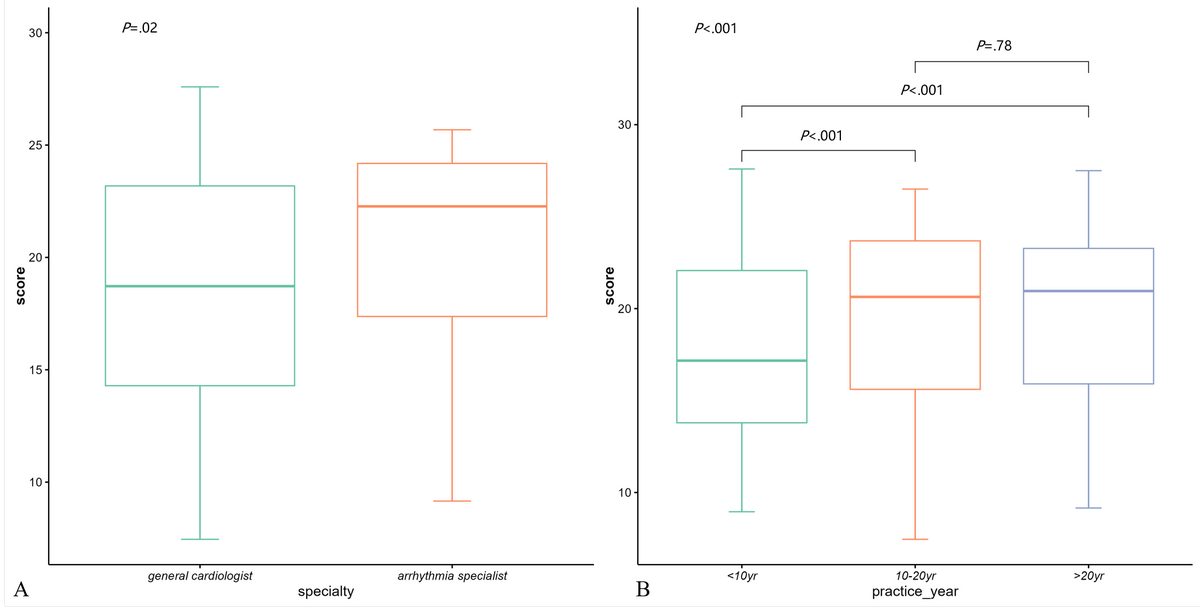
Subgroup analyses according to (A) specialty and (B) years of practice.

We divided all the cardiologists into subgroups based on specialty (Figure 5A) and practice years (Figure 5B). The Kruskal-Wallis test showed a significant difference in practice competency scores between general cardiologists and arrhythmia specialists (*P*=.02). Subgroup analysis according to years of practice revealed a significant effect of experience on scores. The <10 years group had significantly lower scores compared to those of the 10-20 years and >20 years groups. However, there was no significant difference between those with 10-20 years and >20 years of experience.

## Discussion

### Principal Results

Based on a combination of semistructured interviews and questionnaires, this study conducted a large-scale nationwide survey for cardiologists to understand their knowledge and practice competence about VT diagnosis and their requirements for a related CDSS. The results indicated that knowledge and practice support in examination interpretation, etiological diagnosis, and diagnostic evaluation are considered to be essential for a VT diagnosis CDSS. In addition, the vast majority of the cardiologists gave a positive response with respect to the need for a CDSS.

### CDSS Requirements

Previous research on CDSS requirements has primarily relied on methods such as interviews [31,34,35,38,39] and group discussions [30,34,35] to elicit users’ subjective needs. Based on recommendations from clinical experts and medical informatics professionals within our research team, it was acknowledged that certain objective requirements might not be articulated by users during interviews. Consequently, a questionnaire was designed to assess and uncover the requirements that might not have been spontaneously expressed during interviews. Previous studies have used questionnaires to investigate the knowledge, attitudes, and practices of health care professionals in various specific tasks [46-53], providing a basis for our questionnaire approach. To objectively reflect cardiologists’ knowledge and practice deficiencies, we opted to not directly inquire about specific knowledge points but instead used two case scenarios to simulate authentic VT diagnostic situations, which is proven to be an appropriate method to assess practice competence [54]. The survey results endorsed the advantages of this mixed methods approach. The difficulties in VT diagnosis mentioned by the cardiologists during interviews primarily focused on distinguishing wide QRS tachycardias on ECG and identifying the etiology of VT, with no mention of diagnostic evaluation. However, results from the practice section of the questionnaire indicated poorer competence in diagnostic evaluation compared to etiological diagnosis, suggesting that the interviewees were not consciously aware of their weaknesses in diagnostic evaluation during interviews. Currently, there is no unified systematic method for conducting a CDS requirements analysis. While our method of integrating interviews and questionnaires provides a comprehensive approach, there is still room for improvement. Use of a simulation game has been suggested as a better means for clinical competence assessment [42]. Future research could consider incorporating cognitive analysis [55] and real-world system usability evaluation [56] to further optimize CDSS requirements analysis.

The objective results from case simulations also affirmed the cardiologists’ need for decision support (Figure 4). Regarding knowledge requirements, the results from the CDS needs section of the questionnaire indicated that participants had relatively fewer demands for knowledge support compared to direct decision support. Moreover, the cardiologists revealed a preference for automatically prompted relevant knowledge during the diagnostic and therapeutic processes, which can provide more targeted knowledge support (Figure 2). The challenge lies in ensuring that the CDSS accurately identifies the current diagnostic and therapeutic tasks; determines user knowledge gaps; and automatically retrieves, integrates, and presents knowledge support rapidly and accurately [57]. The results of the practice competence highlighted the need for improvement in the interpretation of diagnostic tests, etiological diagnosis, and diagnostic evaluation, suggesting the need for decision support in these three aspects, which were also highlighted as key clinical reasoning [58]. Notably, the accuracy of etiological diagnosis was relatively high, aligning with the lower knowledge demand for an etiological diagnosis (Figure 3). In terms of CDSS needs, the cardiologists favored direct decision support over knowledge support, including explanatory diagnoses and executable evaluation processes, which has also been recognized in recent studies [57,59,60].

Synthesizing the findings of this study, we propose the following recommendations of specific functions of a CDSS for VT diagnosis under a framework of knowledge and practice. With respect to knowledge support, the CDSS needs to (1) provide foundational knowledge by offering fundamental knowledge for each relevant disease that is available for clinicians to retrieve and browse; (2) contextualize knowledge delivery by providing closely related knowledge at decision points, including, but not limited to, the interpretation of diagnostic tests such as ECGs and echocardiograms, wide QRS complex differentiation, etiological diagnosis of VT, and the issuance of diagnostic test orders; (3) explain the knowledge underlying CDSS results; and (4) provide evidence-based recommendations at decision points with available evidence support. With respect to practice support, the CDDS should (1) assist in ECG interpretation, including distinguishing wide QRS complex tachycardias, identifying useful features for etiological diagnosis during sinus rhythm and VT, and recommending diagnostic test orders; (2) assist in echocardiogram interpretation, including the recognition of common etiologies of VT such as old myocardial infarction, ARVC, myocarditis, and the classification of phenotypes of cardiomyopathies; (3) provide suspected etiological diagnoses based on existing information for patients with VT, including acute coronary syndrome, ischemic cardiomyopathy, ARVC, and acute myocarditis, with specific emphasis on alerting clinicians who may not have considered the possibility of acute coronary syndrome; and (4) supplement diagnostic assessments with additional information, including critical medical history, physical examination, laboratory tests, and other examinations. Particularly, using a comprehensive differential diagnosis list is advocated to mitigate premature closure [14], as substantiated by a recent study [61].

Dxplain [24], one of the few DDSSs available for general practice, provides a diagnosis list according to input patient manifestations, which aligns with our proposed structure for VT etiological diagnosis. However, Dxplain lacks knowledge support, examination interpretation, and diagnostic assessment functions, which are highlighted as requirements for a VT CDSS as mentioned above. Another well-known commercial diagnostic support tool, ISABEL, not only serves as a diagnosis reminder but also provides knowledge support (ie, evidence-based knowledge of each disease). However, it does not satisfy the other requirements identified in this study [23,62]. Dr. Mayson [63] is a Chinese commercial CDSS for general practice, which can abstract data from electronic health records to form a diagnosis list as well as provide assistance in diagnostic assessment. Like ISABEL, Dr. Mayson provides a knowledge database for each disease, including clinical practice guidelines. However, the knowledge support is at the disease level rather than the decision level. In addition, this CDSS does not assist with examination interpretation.

Although our study mainly investigated the specific functionalities for VT diagnosis, the results indicated some general CDSS functionalities, including interpretability of decision-making as well as the overall feasibility of the CDSS workflow. Several reviews [64-66] summarized other universal features worthy of consideration, such as integration with the clinical workflow and electronic health record system, reduction of manual input of patient data, execution users’ desired action, avoidance of unnecessary alerts, documentation of reasons for rejecting recommendations, as well as the “five rights” of CDS (providing the right information to the right people in the right formats through the right channels at the right time) [67].

We believe that an excellent CDSS should provide tailored assistance for different types of clinicians. Thus, a subgroup analysis was performed according to the clinician characteristics in the practice section (Figure 5). As anticipated, arrhythmia specialists outperformed general cardiologists, which aligns with the findings of previous research [68]. The American College of Cardiology defines different types of cardiovascular specialists that have requirements for different types of support in cardiovascular health care [69]. A CDSS should be tailored to clinicians’ specialization levels to assist in diagnostic and therapeutic practices. For highly specialized clinicians facing a narrow spectrum of diseases, CDSS assistance may be limited, while support for foundational diagnostic and therapeutic aspects outside their specialty may be necessary. Conversely, less specialized clinicians facing a broader spectrum of diseases may need support in staying updated with the latest diagnostic and therapeutic advancements. For instance, for less experienced clinicians facing patients with VT, the CDSS should always indicate the possibility of CAD. For experienced clinicians, as they have already cultivated the mindset to exclude CAD, the CDSS might only provide this alert when they miss the diagnosis of CAD. Furthermore, it is expected that the CDSS could continually adapt to individual needs through observing clinician users’ behaviors. The impact of years of practice on performance seems to be nonlinear. Clinicians practicing for 10-20 years or more demonstrated better performance than those practicing for less than 10 years. However, there was no significant difference between the 10-20 years and >20 years groups, suggesting that clinical skills may grow in the first 10 years of practice but plateau afterward, thereby challenging the CDSS design to provide targeted support for clinicians with different levels of experience in practice. Additionally, for clinicians entering a bottleneck period in competence growth, the CDSS could facilitate education during practice, thereby supporting lifelong learning. Several studies have been performed in this regard in the areas of pharmaceutical skills [70], imaging interpretation [71], geriatric care [72], and periprocedural antithrombotic use [73].

Most existing CDSSs have been generally designed for health care providers but might not fully consider the diversity of requirements as well as their expertise levels [74]. The genuine needs of health care providers have not been effectively communicated to system developers, resulting in the design of CDSSs that struggle to fulfill their intended role of assistance and workload reduction. Our study centers around the clinical scenario of VT diagnosis, comprehensively exploring support requirements in both knowledge and practice. This investigation can thus provide a foundation for the development of a relevant CDSS. Additionally, we aspire for this study to serve as a reference for clinical needs research, encouraging more health care providers and system developers to scrutinize clinical requirements and establish a groundwork for the development of highly effective CDSSs.

### Limitations

Although this study used a combination of structured interviews and questionnaires for assessment, inevitably, some subjective factors from the participants may have biased the results. The questionnaire content of this study was carefully designed based on the results of the interviews as well as the experience of the multidisciplinary team; however, the questionnaire content was unable to cover all aspects of knowledge and practice related to VT diagnosis. Although specific functions for a VT diagnosis CDSS were proposed, they have not been evaluated in a real-world setting. As our team is currently developing a VT CDSS with these functions, more rigorous studies will be conducted to support these findings in our future research.

### Conclusions

This comprehensive analysis of VT CDSS requirements using a mixed methods approach identified specific knowledge and practice support requirements. The derived functions provide a foundation for further development and optimization of a CDSS. Moreover, it is important to tailor the CDSS to clinicians’ specialization levels and years of practice for effective and personalized support.

## Appendix

Multimedia Appendix 1 Demographic characteristics and clinical experience of the interviewees.

Multimedia Appendix 2 Complete version of the questionnaire with the participant consent form.

## Abbreviations

AHRA: Asian Heart Rhythm Association
ARVC: arrhythmogenic right ventricular cardiomyopathy
CAD: coronary artery disease
CDS: clinical decision support
CDSS: clinical decision support system
DDSS: diagnostic decision support system
ECG: electrocardiogram
ESC: European Society of Cardiology
SCD: sudden cardiac death
VT: ventricular tachycardia

## Data Availability

The data sets generated and/or analyzed during this study are available from the corresponding author on reasonable request.
