# Supplementary material for: Clinical Decision Support Requirements for Ventricular Tachycardia Diagnosis Within the Frameworks of Knowledge and Practice: Survey Study
Source: JMIR Hum Factors. 2024 Mar 26;11:e55802. doi: 10.2196/55802 (PMC11005434; doi:10.2196/55802)
Supplement: Multimedia Appendix 2 [file humanfactors_v11i1e55802_app2.docx]

Complete version of questionnaire and score assignment

Concise Description about the Survey

We are conducting a survey on the knowledge mastery of ventricular tachycardia, aiming to understand the doctors' awareness of ventricular tachycardia, the current status of diagnosis and treatment, and the demand situation, identify the existing problems and causes, and propose corresponding solutions. So we would like to invite you to take a few minutes to fill out this questionnaire. The content of this questionnaire is only for scientific research. No personal and hospital privacy are involved, please fill in truthfully according to your own situation and experience. Note: You can also select an answer for the "Multiple Choice Questions" shown.

Informed consent

If you agree with the above, please start answering the question by clicking “go on” button under the page. If you disagree with the above, please interrupt this survey. Thank you very much for your help and support!

Note: the options in red were the correct answer. The scores were assigned to each option in bracket following.

Basic information

1. Your gender?
2. male
3. female
4. Your age?
5. ≤30 years
6. 31~35 years
7. 36~40 years
8. 41-45 years
9. 45-50 years
10. 51-55 years
11. 56-60 years
12. ≥61 years
13. Your department?
14. family physician
15. cardiovascular
16. arrhythmias and electrophysiology
17. others (please specify)
18. Your title?
19. resident physician
20. attending physician
21. associate chief physician
22. chief physician
23. The year of your practice?
24. <3 years
25. 3-5years
26. 6-10years
27. 10-15years
28. 15-20years
29. >20years
30. The tiers of your hospital?
31. Tertiary A
32. Tertiary B
33. Tertiary C
34. Secondary A
35. Secondary B
36. Secondary C

Knowledge mastering

Case 1

7. A 55-year-old male is admitted to the emergency department due to "intermittent palpitations with chest tightness for 4 years, and recurring 1 hour recent". The patient has hypertension for 10 years and diabetes for 8 years. The ECG is as follows. What is the diagnosis?


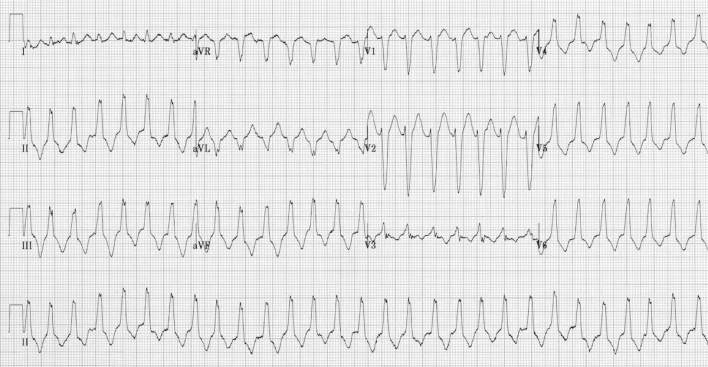


1. sinus tachycardia with left bundle branch block
2. supraventricular tachycardia with left bundle branch block
3. intraventricular conduction delay
4. ventricular tachycardia

If the responder does not choose D, Question 8 will be skipped.

**Intention**: identify ventricular tachycardia in ECG

8. What’s the origin of this patient’s ventricular tachycardia?

1. right ventricular outflow tract
2. right ventricular basal
3. Left ventricular outflow tract
4. left ventricular basal

**Intention**: identify the sites of origin of ventricular tachycardia

9. The patient reports that he had an episode of syncope 2 years ago. Now, some exam results are as followed: cTnI 1.02ng/ml, NT-proBNP 1500pg/ml, echocardiogram: right ventricular end-diastolic diameter enlargement, decreased right ventricular movement, right ventricular aneurysm. The ECG at sinus rhythm is as below. What is the most likely diagnosis?


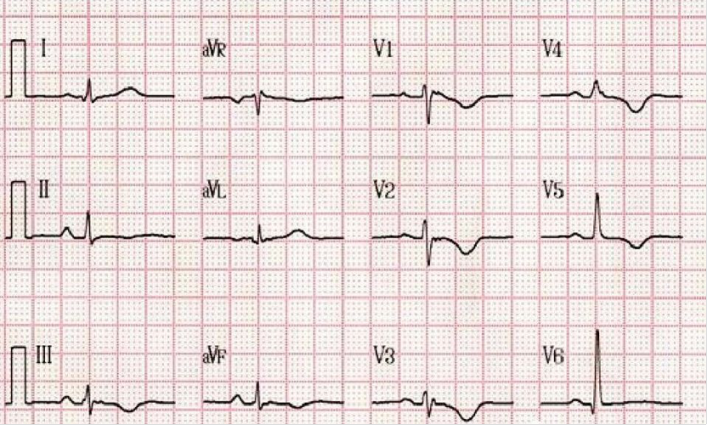


1. idiopathic right ventricular tachycardia
2. prior myocardial infarction
3. Brugada syndrome
4. arrhythmogenic right ventricular cardiomyopathy
5. early repolarization syndrome

**Intention**: the ECG and echocardiographic manifestations of ARCV, which is a main differential diagnosis about right ventricular tachycardia.

10. To further assist in diagnosis, which of the following examinations should order preferentially:

1. coronary angiography
2. holter
3. intracardiac electrophysiology examination
4. cardiovascular magnetic resonance imaging
5. endomyocardial biopsy

**Intention**: the guideline recommended evaluation of ARVC; the strategy of exclusion of coronary heart disease when encountering ventricular tachycardia.

case2

11. A 35-year-old female complains about "fever and fatigue for 1 week, chest pain for 1 hour". The patient has been of high fever 1 week ago. She has taken oral cephalosporin antibiotics, but it has no effect. She also has chest pain 1 hour ago, with nausea, vomiting, clammy skin and oliguria. Physical examination: body temperature 38.0 °C, breathing 39 times/min, BP 70/49mmHg; poor response, paleness, low dull heart sounds, abdominal pain, liver enlargement. cTnI 5.12 ng/ml, Echocardiography: ventricular wall diffuse hypokinesia, LVEF30%, The electrocardiogram is as followed. What diagnosis needs to be considered?


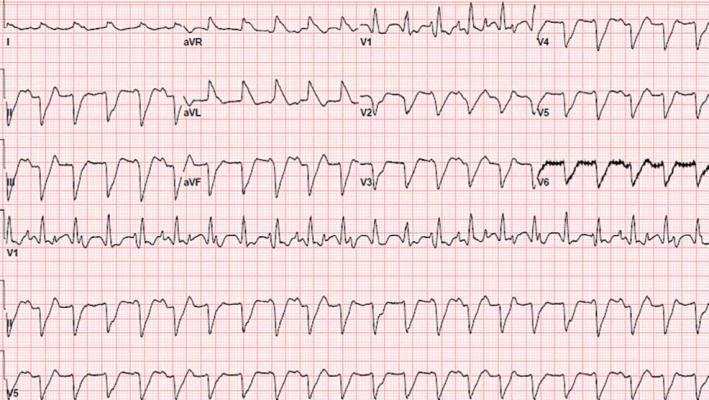


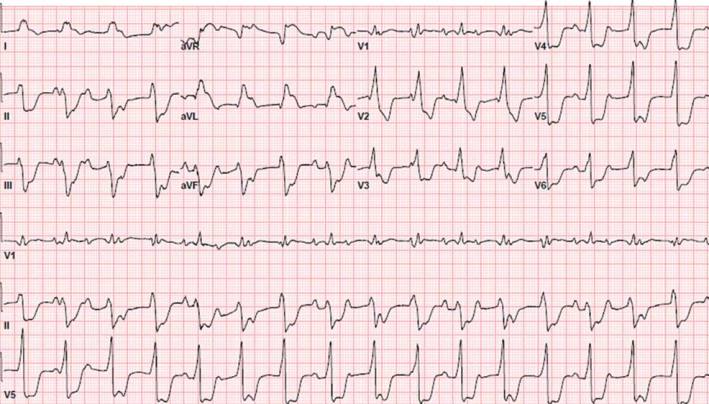


1. acute heart failure
2. acute myocardial infarction
3. acute myocarditis
4. acute pericarditis
5. acute aortic dissection
6. acute abdomen

If the answer of Question 11 contains B, it will go on to Question 12, otherwise it will go no to Question 13.

**Intention**: the manifestation of acute myocarditis; the alert about coronary heart disease when encountering ventricular tachycardia.

12. The urgent coronary angiography shows that there is no significant stenosis in the left main trunk (LM), left anterior descending branch (LAD), left circumflex branch (LCX), and right coronary artery (RCA). Subsequently, antibiotics, intravenous fluids and vasopressors are given, but the patient's condition does not improve. What treatment should be taken next ?

1. extracorporeal membrane oxygenation (ECMO)
2. continuous renal replacement therapy (CRRT)
3. dose up intravenous fluids and vasopressors therapy
4. glucocorticoid therapy
5. heart transplant

**Intention**: the identification and treatment of fulminant myocarditis

13. The patient then has no consciousness, no breathing , and the blood pressure cannot be measured. The patient is rescued temporarily from cardiopulmonary resuscitation and advanced cardiac life support. What management should be taken next ?

1. extracorporeal membrane oxygenation (ECMO)
2. continuous renal replacement therapy (CRRT)
3. urgent coronary angiography
4. percutaneous coronary intervention or coronary artery bypass grafting
5. glucocorticoid therapy
6. heart transplant

**Intention**: the alert about coronary heart disease when encountering ventricular tachycardia, especially there is a sudden cardiopulmonary condition worsening.

14. As the <2022 ESC guidelines for the management of patients with ventricular arrhythmias and the prevention of sudden cardiac death> suggests a protocol for managing patients with the first presentation of sustained monomorphic ventricular tachycardia, which parts of the protocol you lack knowledge in?


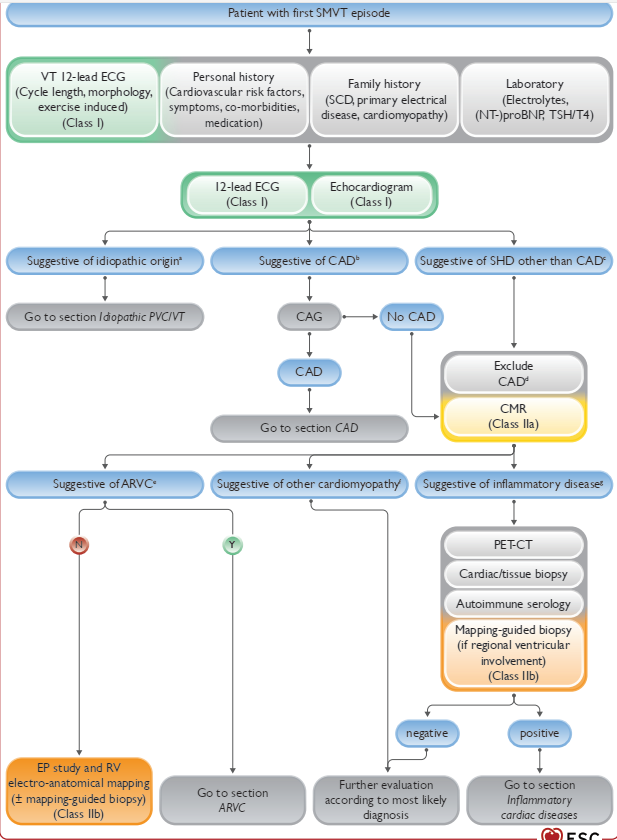


1. Examination interpretation

- Fully applies
- Rather applies
- Rather doesn’t apply
- Doesn’t apply at all

1. Etiological diagnosis

- Fully applies
- Rather applies
- Rather doesn’t apply
- Doesn’t apply at all

1. Diagnostic evaluation

- Fully applies
- Rather applies
- Rather doesn’t apply
- Doesn’t apply at all

1. Conceptual knowledge

- Fully applies
- Rather applies
- Rather doesn’t apply
- Doesn’t apply at all

Clinical decision support needs

15. Have you ever heard of clinical decision support system that assists in the diagnosis and treatment of ventricular arrhythmias or myocardial diseases?

1. Yes
2. No

16. Have you ever used clinical decision support system that assists in the diagnosis and treatment of ventricular arrhythmias or myocardial diseases?

1. Yes
2. No

17. Do you wish for a clinical decision support system that assists in the diagnosis and treatment of ventricular arrhythmias or myocardial diseases?

1. Yes
2. No

18. What functions you wish for the clinical decision support system?

1. Interpretable diagnosis (suggest possible diagnosis and supporting evidence)

- Fully applies
- Rather applies
- Rather doesn’t apply
- Doesn’t apply at all

1. Executable processes (suggest history and auxiliary examinations still required)

- Fully applies
- Rather applies
- Rather doesn’t apply
- Doesn’t apply at all

1. Knowledge support (provide pertinent knowledge from textbooks/guidelines/literatures)

- Fully applies
- Rather applies
- Rather doesn’t apply
- Doesn’t apply at all
